# A BERT Framework to Sentiment Analysis of Tweets

**DOI:** 10.3390/s23010506

**Published:** 2023-01-02

**Authors:** Abayomi Bello, Sin-Chun Ng, Man-Fai Leung

**Affiliations:** School of Computing and Information Science, Faculty of Science and Engineering, Anglia Ruskin University, Cambridge CB1 1PT, UK

**Keywords:** sentiment analysis, deep learning, tweets, BERT, LSTM, CNN

## Abstract

Sentiment analysis has been widely used in microblogging sites such as Twitter in recent decades, where millions of users express their opinions and thoughts because of its short and simple manner of expression. Several studies reveal the state of sentiment which does not express sentiment based on the user context because of different lengths and ambiguous emotional information. Hence, this study proposes text classification with the use of bidirectional encoder representations from transformers (BERT) for natural language processing with other variants. The experimental findings demonstrate that the combination of BERT with CNN, BERT with RNN, and BERT with BiLSTM performs well in terms of accuracy rate, precision rate, recall rate, and F1-score compared to when it was used with Word2vec and when it was used with no variant.

## 1. Introduction

It is obvious that the emergence of real-time information networking platforms such as Twitter has led to the development of an unmatched public collection of viewpoints about all relevant worldwide entities thereby interfering and affecting human lifestyle [1]. Twitter may be a great platform for opinion generation and presentation, but it also presents new and unique obstacles, and the process would be incomplete without capable tools for assessing those thoughts to speed up their consumption. The best approach over time has proven to be using sentiment analysis tools to identify individual attitudes and emotions [2].

Sentiment analysis, which is additionally referred to as subjective investigation or artificial intelligence of emotions) is a natural language processing (NLP) technique for extracting information patterns and key characteristics from a large body of text. It analyses the thoughts, attitudes, viewpoints, opinions, convictions, remarks, requests, inquiries, and interests expressed by the author based on feelings not reason in the form of texts, with entities such as service, issue, person, product, event, object, organizations, and their attributes. It identifies the author’s overall attitudes toward a text, which could be anything from blog posts to product reviews to online forums to speeches to data from databases to social media documents [3].

The need for natural language processing (NLP) arises as a result of the need for computers to understand the spoken and written language of humans. This brought about bag of words (BOW) which uses N-grams but the contextual meaning of words is ignored with BoW models, after which the word embedding was developed to overcome this issue which always takes words similar in meaning as similar contexts but this model always relies on large vocabulary and high computational power [4]. Word2Vec was then developed which generates only one vector embedding for each word but also has a shortcoming by considering left or right context. The transformer model which was introduced by Google in 2018 then solves the aforementioned problem and enhances language processing. It helped to overcome the problem of transfer learning and has recorded great achievement on natural language processing tasks such as named entity recognition (NER) and question answering and sentiment analysis. The BERT has been pre-trained on large corpus of English data which acts like a benchmark and helps solve similar problems. The BERT has transformer encoder layers enhanced with a self-attention mechanism.

This study seeks to produce an approach that can identify the opinion and attitude of a writer in a tweet according to context. The pre-trained transformer BERT and Word2Vec was used with the convolutional neural network (CNN), recurrent neural network (RNN), bidirectional long short-term memory (BiLSTM), and the experiment was carried out and the Proposed BERT obtained a state-of-the-art performance.

This study’s contributions include proposing the BERT for NLP that can identify sentiment in tweets according to three categories (positive, negative, and neutral) based on the context of the writer. Our approach is distinct from similar studies because:

- We have trained our model using six different datasets which is a combination of different tweets.
- We combined knowledge embedded in the pre-trained bidirectional transformer (BERT) with a deep learning classifier to detect sentiment (positive, negative, or neutral) other than just using a machine learning classifier.
- The proposed BERT will dynamically generate a vector according to the word context and when placed into deep learning classifiers such as CNN, RNN, or BiLSTM to predict output, achieves an accuracy of 93% and F-measure of 95%.

## 2. Literature Review

The development of the internet has changed how people now express their ideas and thoughts. According to Kepios, there are 4.74 billion social media users around the world equating to 59.3 percent of the total global population. For context, the data suggest that more than 75 percent of the eligible global population now uses social media [5]. Further research shows that a typical social media user actively uses or visits an average of 7.2 million different social platforms each month and spends close to 2 and half hours per day using social media. The world spends more than 10 billion hours using social platforms each day which is equivalent to nearly 1.2 million years of human existence. Additionally, social media gives businesses a chance by offering them a platform to engage with their customers for advertising. People heavily rely on user-generated content from the internet when making decisions. Social networking services such as Twitter are a valuable source of information to find out what happened or what is happening in a geographic area [6]. Microblogs have become an important origin of information regarding events happening in a location during a period of time [7]. Twitter is one of the most used platforms with easy access to tweets with connection to the API and having a maximum length of 280 characters making it suitable to effectively monitor emotions, sentiments, opinions, and attitudes of different subject matter.

Artificial Intelligence (AI) is the art and science of building intelligent machines, particularly smart computer programs. Furthermore, AI can be defined as the imitation or reproduction of cognitive functions by computer systems that can reason logically and act in ways resembling those of humans. The subject gained popularity as a subject in academic literature after the 1950s. Various industries use AI, including communication, IT, healthcare, agriculture, logistics, education, and aviation.

In recent years, natural language processing (NLP) has drawn a lot of interest for its ability to computationally represent and analyze human language. It has expanded the range of industries in which it is used, including machine translation, email spam detection, information extraction, summarization, and medicine [8]. It makes interactions between people and computers simple and effective using computational linguistics and machine learning. NLP systems can output written texts or processed speech from inputs such as text, images, or speech [9].

Neural network is a subset of machine learning with numerous applications such as compressed image reconstruction [10], asset allocation [11,12], non-negative matrix factorization [13,14], and model predictive control [15]. With the development of transfer learning, G.E. Hinton introduced the concept of deep learning, and it is simply extracting features from raw data with the help of using layers [16]. The human brain affects neural networks, which are made up of numerous neurons and form amazing networks. Deep learning networks can be used to teach both supervised and unsupervised categories [17,18,19,20]. CNN, RNN, and many other networks with more than three layers are considered deep learning approaches. Text creation, vector representation, word representation estimation, sentence classification, phrase modeling, feature presentation, and emotion recognition benefit greatly from neural networks [21,22].

Additionally, the term deep learning has gained popularity among computer scientists to refer to pattern-recognition algorithms that enable computers to learn on their own, leading to improvements in speech and image recognition as well as more precise translation software. In addition to a deeper focus on context, thought, and abstraction, deep learning can also refer to knowledge that is less surface level and more contemplative and abstract [23].

### 2.1. Sentiment Analysis Based on Machine Learning Approach

Suhasini et al. [4] were able to identify emotions on Twitter using supervised learning. K-nearest neighbor (KNN) and naive Bayes (NB) were the two algorithms compared and the study shows that naive Bayes outperformed the K-nearest neighbor.

Jayakody et al. [1] collected data from twitter posts based on product review, then analyzed using the support vector machine (SVM), logical regression, and K-nearest neighbor machine learning algorithm and count vectorizer and term frequency-inverse document frequency mechanisms for converting text into vectors for the data to be inputted into the machine learning model. The highest accuracy score was achieved by logistic regression with a count vectorizer with an accuracy rate of 88.26%.

Bhagat et al. [24] used a hybrid approach of naive Bayes and K-nearest neighbor to divide tweets into three classes: positive, negative, and neutral, and they achieved a better accuracy than the random forest.

### 2.2. Sentiment Analysis Based on Deep Learning and BERT Approach

Chiorrini et al. [25] proposed two BERT-based approaches for text classification: BERT-base and cased BERT-base. They gathered information from microblogging sites, particularly twitter. Two separate datasets were employed in their experiment, and they were used for sentiment analysis and emotion recognition. The proposed model gives an accuracy of 92%. They emphasized that BERT produces positive outcomes for text classification.

Huang et al. [26] presented a model for text classification where he used the deep convolutional neural network, bidirectional gated recurrent. This model was based on BERT. Two datasets (CCERT email and movie comment) were used. The result gave an accuracy of 92.66% on CCERT and 91.89% on the movie dataset.

The researchers of [27] represented a seven-layer framework to analyze the feelings of sentences. This framework was based on CNN and Word2vec to calculate vector representation and SA, respectively. Google has proposed the use of Word2vec. To improve the correctness and generalizability of the suggested model, the researcher employed the dropout technology, normalization, and parametric rectified linear unit (PReLU). The framework was validated using a data set from rottentomatoes.com that contains a corpus of movie review extracts with five labels: positive, slightly positive, neural, negative, and somewhat negative. Compared to earlier models, such as matrix-vector recursive neural network (MV-RNN) and recursive neural network, the suggested model outperformed the previous with an accuracy of 45.4%.

## 3. Materials and Methods

This section provides a concise and precise description of the experimental results, their interpretation, as well as the experimental conclusions that can be drawn.

### 3.1. Data Collection and Preprocessing

**Figure 1:**
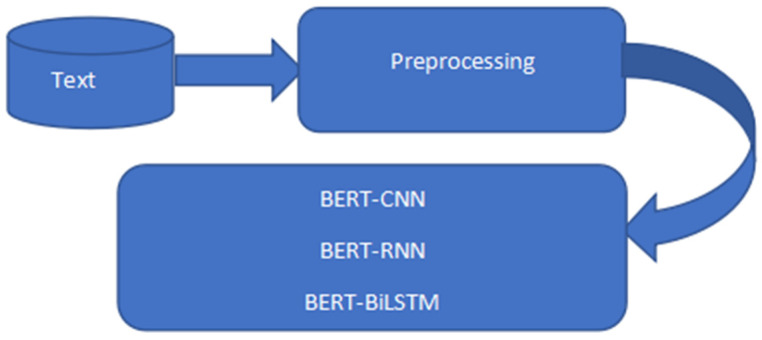
BERT-based architecture.

**Figure 2:**
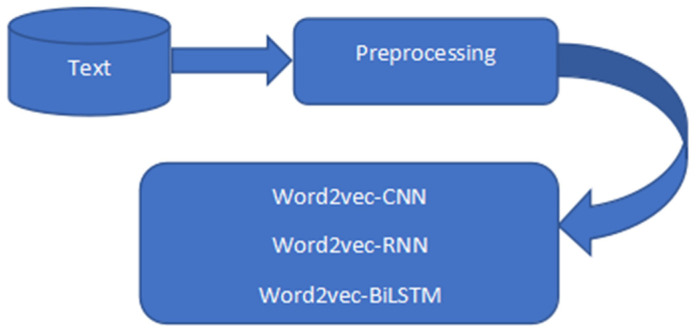
Word2Vec-based architecture.

**Figure 3:**
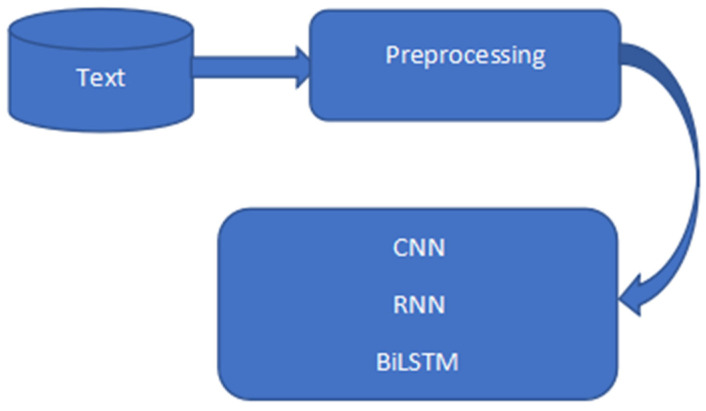
CNN, RNN, BiLSTM architecture.

Six datasets were used and they were tweets collected from Kaggle. The six datasets were combined using Python’s concatenating function. There are 2 columns and 212,661 rows in the dataset altogether. The null values were eliminated, and mapping was completed. 1.0 was assigned to positive, 0.0 to neutral, and −1.0 to negative. Special characters, punctuation, numbers, symbols, and hashtags were removed from the model dataset as shown in Table 1 below. The dataset sentiment contains 71,658 neutrals, 85,231 positives, and 55,772 negatives and has a percentage of 40.1% to be positive, 33.7% neutral, and 26.2% negative as shown in Table 2 below. The raw tweets were preprocessed and fed into different models as shown in Figure 1, Figure 2 and Figure 3. 

### 3.2. Bidirectional Encoder Representations from Transformers (BERT)

BERT was developed in 2018 for natural language understanding tasks to assist machines in comprehending the context of phrases [28]. It employs transfer learning, and the architecture is based on the transformer model. Transfer learning entails training a model for a broad task and then applying the knowledge gained to fine-tune BERT for a new task [29]. BERT has been trained on two tasks: masked language tasks, in which sentences are fed to the model and some words are masked or hidden for the model, and the model attempts to predict these hidden words, and unmasked language tasks, in which sentences are fed to the model and some words are masked or hidden for the model, and unmasked language tasks, in the other task is sentence prediction where pair of sentences are fed to the model each round and the model need to predict whether one sentence is followed by the other or not. BERT has been trained over a large dataset for these two tasks. The dataset contained all English Wikipedia and 11,038 books. BERT uses an encoder from a transformer model, a type of neural network that takes a sentence as input to the model. Then each word of the sentence is tokenized, and these tokenized words are fed to the BERT model. BERT output is a vector representation for each tokenized word.

Using encoders from Transformer enables BERT to have a better context understanding than traditional neural networks such as LSTM or RNN since the encoder process all inputs, which is the whole sentence, simultaneously so when building a context for a word, BERT will take into account the inputs before it and also the inputs after the word, while the LSTM or RNN process the input taking in account only the prior inputs, and that will be reflected on the output vector value for the word, so the word “python” in the two sentences (I just started learning Python) and (Python are found in part of Africa and Asia) would have the same vector value—and as a result the same meaning—when using LSTM or RNN; on the other hand, it would have two different vectors using BERT, so as a result, using BERT will in most cases give us better performance than using the traditional machine learning algorithms.

### 3.3. Word Embedding

Computers are programmed to operate in numbers. It worked on Bits which are zeros and ones. The question now is what happens when the software or a task must process a word? This word needs to be given to the computer as the only thing it can understand is numbers which means it needs to be broken down into bits (zeros and ones). The most straightforward approach in NLP is to create a vocabulary with many terms and assign a number to each word in the vocabulary [30]. Word embedding analysis is a natural language processing method in which a neural network is trained using machine learning to anticipate the contexts in which words are employed [28].

### 3.4. RNN

RNN is a type of artificial neural network that identifies patterns in data and utilizes them to anticipate the following most likely outcome. It operates on the tenet that each layer’s output is saved and fed back into the system’s input in order to forecast that layer’s output.

For the RNN, the learning rate was set to be 0.01, on 10 epochs using Adam optimizer with batch size of 128, activation was softmax and categorical crossentropy was used as the loss as shown in Table 3 below.

### 3.5. CNN

CNN is formed of different layers of neurons. This works best on images, when an image is entered, each layer of the network creates a number of activations that are passed on to the following layer. Typically, the first layer extracts fundamental features such as edges that run horizontally or diagonally. The following layer receives this output and detects more intricate features such as corners or multiple edges. The network may recognize increasingly more complex elements, including objects, faces, etc., as we go further into it. 

For the CNN, the model was trained on 10 epochs using Adam optimizer with batch size of 128, the activation was softmax and categorical crossentropy was used as the loss as shown in Table 4 below.

### 3.6. BiLSTM

A bidirectional long short-term memory, or BiLSTM, was employed since it is a model in which processing is done in order. It comprises dual LSTMs, one is open to input in one direction, while the other is open to input in another. A fully connected neural network is made up of multiple fully connected layers that link all of the neurons in each layer to all of the neurons in the other layer [31].

For the BiLSTM, the model was trained on 10 epochs using Adam optimizer with batch size of 128, activation was softmax and categorical crossentropy was used as the loss as shown in Table 5 below.

### 3.7. Word2Vec

For each word, the Word2Vec embedding approach only offers a single, independent embedding vector. Only one vector per word is saved by Word2vec in the output model. Although Word2vec is trained using contextual neighbors, a downstream NLP task uses it without context, since the representation is only kept as one vector per word. Therefore, stagnant in use. This restricts the ability to understand a word’s meaning across two contexts and used in two different situations, for example, “river bank” and “bank deposit”, “apple macbook” and “apple as a fruit” or “Python” as a programming language and “python” as a snake.

For the Word2vec with CNN, RNN and BiLSTM, the model was trained on 10 epochs with learning rate 1 × 10^−5^ using Adam optimizer with batch size of 32, activation was softmax and categorical crossentropy was used as the loss as shown in Table 6 below.

### 3.8. BERT

BERT has been trained on two tasks: masked language tasks, in which sentences are fed to the model and some words are masked or hidden for the model, and the model attempts to predict these hidden words, and unmasked language tasks, in which sentences are fed to the model and some words are masked or hidden for the model, and unmasked language tasks, in the other task is sentence prediction where pair of sentences are fed to the model each round and the model need to predict whether one sentence is followed by the other or not. BERT has been trained over a large dataset for these two tasks. The dataset contained all English Wikipedia and 11,038 books. BERT uses an encoder from a transformer model, a type of neural network that takes a sentence as input to the model. Then each word of the sentence is tokenized, and these tokenized words are fed to the BERT model. BERT output is a vector representation for each tokenized word.

Using encoders from Transformer enables BERT to have a better context understanding than traditional neural networks such as LSTM or RNN since the encoder process all inputs, which is the whole sentence, simultaneously so when building a context for a word, BERT will take into account the inputs before it and also the inputs after the word, while the LSTM or RNN process the input taking in account only the prior inputs, and that will be reflected on the output vector value for the word, so the word “python” in the two sentences (I just started learning Python) and (Python are found in part of Africa and Asia) would have the same vector value—and as a result the same meaning—when using LSTM or RNN; on the other hand, it would have two different vectors using BERT, so as a result, using BERT will in most cases give us better performance than using the traditional machine learning algorithms. The BERT comes in two forms which are the BERT base and the BERT large. The BERT base consists of 12 encoders with a hidden size of 768 and the BERT large has 24 encoders with a hidden size of 1024. The study employed the BERT base.

For the BERT with CNN, RNN and BiLSTM, the model was trained on 10 epochs with learning rate 1 × 10^−5^ using Adam optimizer with batch size of 128, the activation was softmax and categorical crossentropy was used as the loss as shown in Table 7 below.

## 4. Results and Discussion

### 4.1. Performance Indicators

Precision is a measure of correctness that explains how many total positive predictions are positive. It is determined by dividing the total number of predicted positives by the total number of classified positives. The precision level should be high for a well-performed model. Precision is defined as follows:

Precision = TP/(TP + FP),(1)

where TP is true positive and FP is false positive.

A recall is the ratio of all positively classified classes that were correctly identified to all positively classified classes or the number of classes with a positive outcome that are correctly predicted. A good model should have a high recall rate. Recall is defined as follows:

Recall = TP/(TP + FN),(2)

where FN is false negative.

A high F1-score indicates high precision and recall because the score contains information about these two variables. It is defined as follows:

F1 = (2 × Precision × Recall)/(Precision + Recall).(3)

Mean absolute error describes the discrepancy between actual and anticipated values. As the value goes down, the model’s performance gets better. An ideal predictor of the outputs is a model with a mean absolute error of zero.

The average of the square of the difference between the data’s original and anticipated values is used to calculate the mean square error. As the value goes down, the model’s performance gets better.

The standard deviation of the errors that result from making a prediction on a dataset is known as the root mean square error. However, when determining the model’s accuracy, the value’s root is considered. As the value goes down, the model’s performance gets better.

### 4.2. Experimental Results

Table 8 shows the summary of the accuracy, precision, recall, and F1-score of all the models considered in the study.

Table 9, below, shows the mean absolute error, mean squared error, and root mean square error of all the models considered in the study.

**Figure 4:**
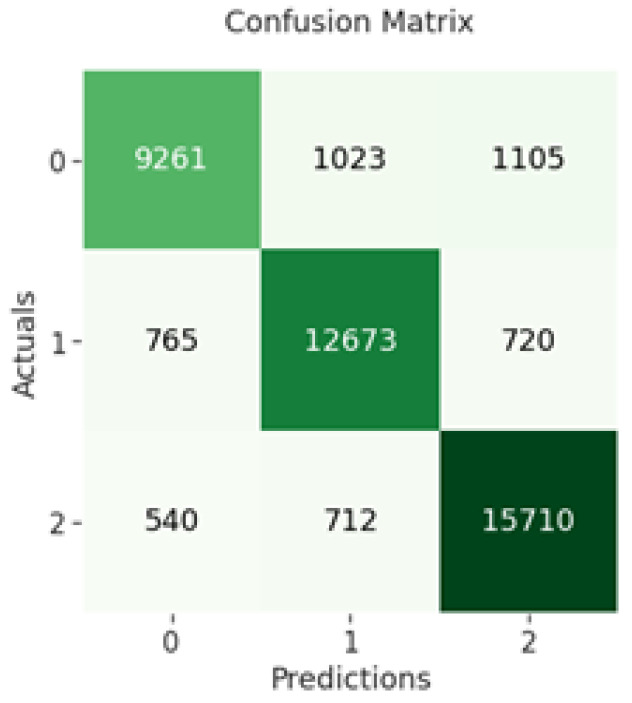
CNN confusion matrix.

The confusion matrix of CNN indicates that 9261 out of 11,389 negative sentiments were correctly classified, while 12,673 out of 14,158 neutral sentiments were correctly classified and 15,710 out of 16,962 positive sentiments were correctly classified as shown in Figure 4 below.

**Figure 5:**
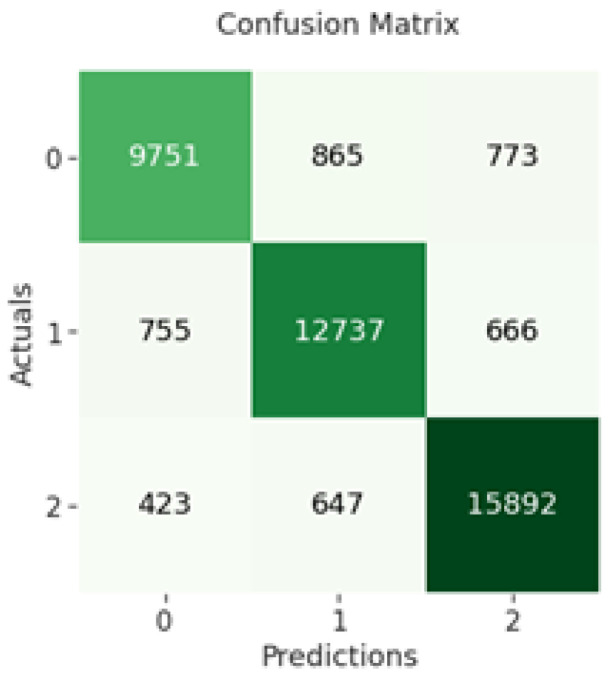
RNN confusion matrix.

The confusion matrix of CNN indicates that 9751 out of 11,389 negative sentiments were correctly classified, while 12,737 out of 14,158 neutral sentiments were correctly classified and 15,892 out of 16,962 positive sentiments were correctly classified as shown in Figure 5 below.

**Figure 6:**
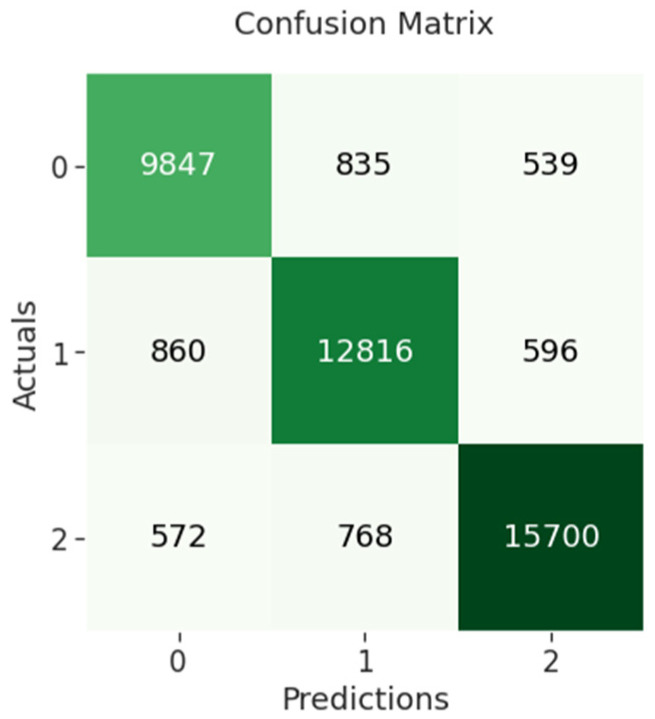
BiLSTM confusion matrix.

The confusion matrix of BiLSTM indicates that 9847 out of 11,221 negative sentiments were correctly classified, while 12,816 out of 14,272 neutral sentiments were correctly classified and 15,700 out of 17,040 positive sentiments were correctly classified as shown in Figure 6 below.

**Figure 7:**
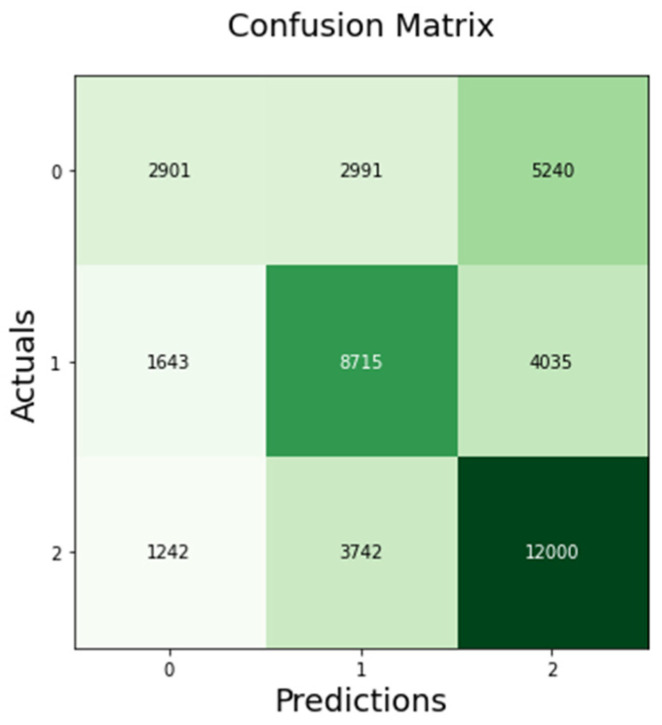
Word2Vec-BiLSTM confusion matrix.

The confusion matrix of Word2Ve-BiLSTM indicates that 2901 out of 11,132 negative sentiments were correctly classified, while 8715 out of 14,393 neutral sentiments were correctly classified and 12,000 out of 16,984 positive sentiments were correctly classified as shown in Figure 7 below.

**Figure 8:**
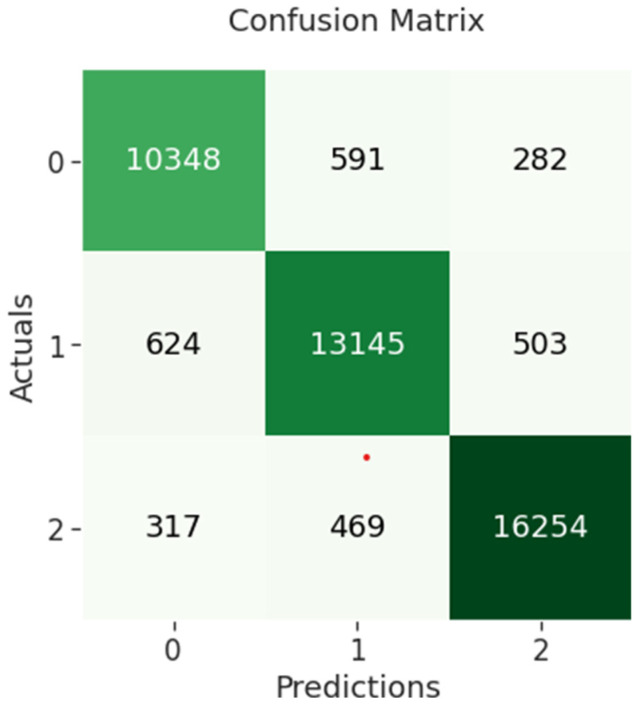
BERT-CNN confusion matrix.

The confusion matrix of BERT-CNN indicates that 10,348 out of 11,221 negative sentiments were correctly classified, while 13,145 out of 14,272 neutral sentiments were correctly classified and 16,254 out of 17,040 positive sentiments were correctly classified as shown in Figure 8 below.

**Figure 9:**
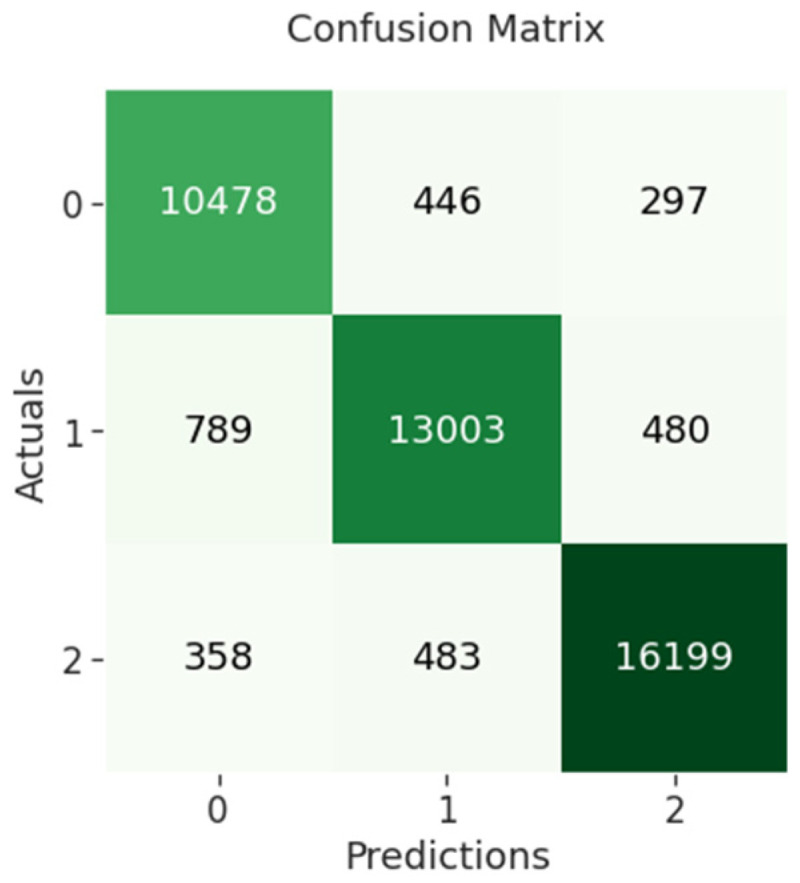
BERT-RNN confusion matrix.

The confusion matrix of BERT-RNN indicates that 10,478 out of 11,221 negative sentiments were correctly classified, while 13,003 out of 14,272 neutral sentiments were correctly classified and 16,199 out of 17,040 positive sentiments were correctly classified as shown in Figure 9 below.

**Figure 10:**
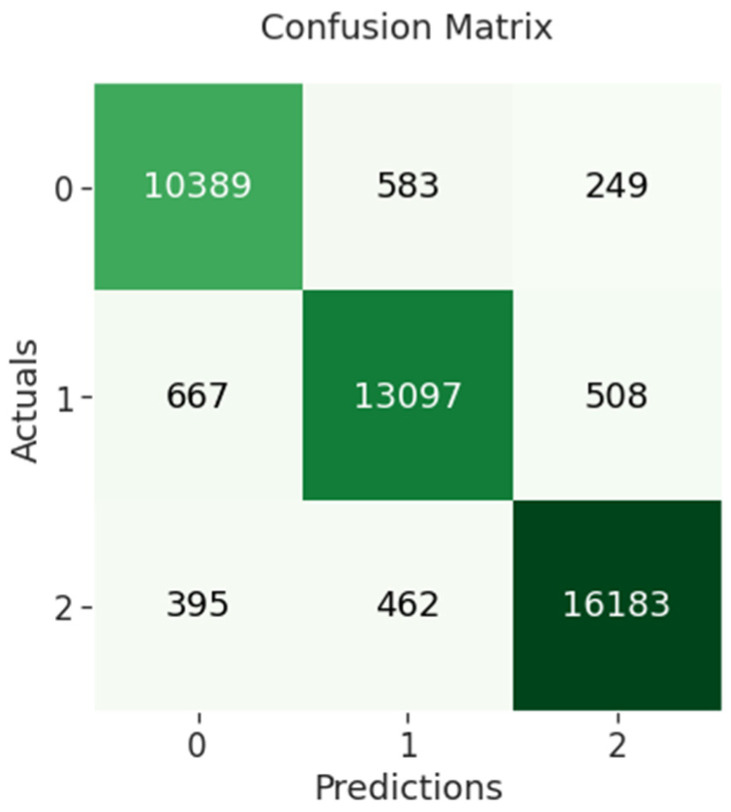
BERT-BiLSTM confusion matrix.

The confusion matrix of BERT-BiLSTM indicates that 10,389 out of 11,221 negative sentiments were correctly classified, while 13,097 out of 14,272 neutral sentiments were correctly classified and 16,183 out of 17,040 positive sentiments were correctly classified as shown in Figure 10 below.

## 5. Conclusions

The traditional approach of natural language processing (NLP) with the use of Word2Vec, CNN, RNN, and BiLSTM has a few limitations of not capturing the deeper context of the word. The BERT has more understanding than traditional since the encoder process all inputs, which is the whole sentence, simultaneously so when building a context for a word, BERT will take into account the inputs before it and also the inputs after the word, while the Word2Vec restricts the ability to understand a word’s meaning across two contexts and used in two different situations which in turn will be reflected on the output vector value for the word.

The combination of the transformer model BERT with CNN, RNN, and BiLSTM gives a state-of-the-art performance in terms of accuracy, recall, and precision.

Further works can be carried out to analyze sentiment on more data that is not sourced online because some information shared online can be shared by tourists or people with little or no understanding about the subject matter. Moreover, instead of sentiment, emotions such as happy, sad, and surprised can also be studied. Other transformer models such as RoBERTa can also be further investigated in further studies.

## Figures and Tables

**Table 1:** Dataset composition.

Dataset Name	Rows by Columns	Source
Twitter_Data.csv	162,980 × 2	Kaggle.com
Apple-twitter-sentiment-texts.csv	1630 × 2	Kaggle.com
FinalSentimentdata2.csv	3090 × 2	Kaggle.com
Tweets.csv	14,640 × 2	Kaggle.com
Train.csv	27,481 × 4	Kaggle.com
Test.csv	3534 × 3	Kaggle.com
Model Dataset after Concatenating	213,355 × 2	

**Table 2:** Sentiment count.

Sentiment	Sentiment Count	Sentiment Percentage
Negative	55,717	26.2%
Neutral	71,658	33.7%
Positive	85,167	40.1%

**Table 3:** Parameter setting of RNN.

Parameter	Values
Learning rate	0.001
Epoch	10
Optimizer	Adam
Batch size	128
Activation	Softmax
Loss	categorical_crossentropy

**Table 4:** Parameter setting of CNN.

Parameter	Values
Epoch	10
Optimizer	Adam
Batch size	128
Activation	Softmax
Loss	categorical_crossentropy

**Table 5:** Parameter setting of BiLSTM.

Parameter	Values
Epoch	10
Optimizer	Adam
Batch size	128
Activation	Softmax
Loss	categorical_crossentropy

**Table 6:** Parameter setting of Word2vec with CNN, RNN, and BiLSTM.

Parameter	Values
Learning rate	1 × 10^−5^
Epoch	10
Optimizer	Adam
Batch size	32
Activation	Softmax
Loss	categorical_crossentropy

**Table 7:** Parameter setting for BERT with CNN, RNN, and BiLSTM.

Parameter	Values
Learning rate	1 × 10^−5^
Epoch	10
Optimizer	Adam
Batch size	128
Activation	Softmax
Loss	sparse_categorical_crossentropy

**Table 8:** Model performance summary.

Models	Accuracy	P	P	P	R	R	R	F1	F1	F1
		NEG	NEU	POS	NEG	NEU	POS	NEG	NEU	POS
CNN	89%	0.88	0.88	0.90	0.81	0.90	0.93	0.84	0.89	0.91
RNN	90%	0.89	0.89	0.92	0.86	0.90	0.94	0.87	0.90	0.93
BiLSTM	90%	0.87	0.89	0.93	0.88	0.90	0.92	0.88	0.89	0.93
Word2Vec-CNN	57%	0.50	0.60	0.60	0.45	0.53	0.70	0.47	0.56	0.64
Word2Vec-RNN	48%	0.50	0.47	0.48	0.30	0.40	0.67	0.37	0.43	0.56
Word2Vec-BiLSTM	55%	0.50	0.56	0.56	0.26	0.61	0.71	0.34	0.58	0.63
BERT-CNN	93%	0.92	0.93	0.95	0.92	0.92	0.95	0.92	0.92	0.95
BERT-RNN	93%	0.90	0.93	0.95	0.93	0.91	0.95	0.92	0.92	0.95
BERT-BiLSTM	93%	0.91	0.93	0.96	0.93	0.92	0.95	0.92	0.92	0.95

**Table 9:** Error evaluation.

Models	Mean Absolute Error	Mean Squared Error	Root Mean Square Error
CNN	0.1531	0.2305	0.4801
RNN	0.1253	0.1815	0.4260
BiLSTM	0.1242	0.1764	0.4200
Word2Vec-CNN	0.2822	0.2822	0.5313
Word2Vec-RNN	0.3456	0.3456	0.5879
Word2Vec-BiLSTM	0.2963	0.2963	0.5443
BERT-CNN	0.0796	0.1078	0.3283
BERT-RNN	0.0825	0.1133	0.3366
BERT-BiLSTM	0.0824	0.1128	0.3358

## Data Availability

Publicly available datasets were analyzed in this study. These data can be found in the following URLs: https://www.kaggle.com/datasets/saurabhshahane/twitter-sentiment-dataset?select=Twitter_Data.csv (accessed on 30 September 2022). https://www.kaggle.com/datasets/seriousran/appletwittersentimenttexts?select=apple-twitter-sentiment-texts.csv (accessed on 30 September 2022). https://www.kaggle.com/datasets/surajkum1198/twitterdata?select=finalSentimentdata2.csv (accessed on 30 September 2022). https://www.kaggle.com/datasets/yasserh/twitter-tweets-sentiment-dataset?select=Tweets.csv (accessed on 30 September 2022). https://www.kaggle.com/code/toygarr/contextual-model-and-crawling-for-real-tweets/data?select=train.csv (accessed on 30 September 2022). https://www.kaggle.com/code/toygarr/contextual-model-and-crawling-for-real-tweets/data?select=test.csv (accessed on 30 September 2022).
